# Supplementary material for: MetaRibo-Seq measures translation in microbiomes
Source: Nat Commun. 2020 Jun 29;11:3268. doi: 10.1038/s41467-020-17081-z (PMC7324362; doi:10.1038/s41467-020-17081-z)
Supplement: Supplementary file 10 — Supplementary Data 7 [file 41467_2020_17081_MOESM10_ESM.zip › File2/Confidence_High_Taxonomy/372810_out.krona.html]

Javascript must be enabled to view this page.

members
magnitude
magnitudeUnassigned
count
unassigned
taxon
rank

372810\_out

2

2
2
superkingdom

2
phylum
1239

186801
class
2

2
order
186802

family
2
541000

946234
2
genus

1193534

SRS049402\_contig\_number\_contig-100\_700.106997SRS148511\_contig\_number\_3956
2
species
